# Conversion of apoptosis to necrosis and the corresponding alteration in the oxidative milieu of male germ cells of rat under acute heat stress: An experimental study

**Published:** 2018-09

**Authors:** Kuldeep Kaushik, Naveen Kaushal, Natwar Raj Kalla

**Affiliations:** 1 *Department of Zoology, Panjab University, Chandigarh, India.*; 2 *Department of Biophysics, Punjab University, Chandigarh, India.*

**Keywords:** Heat stress, Apoptosis, Necrosis, Antioxidant enzymes, Testes

## Abstract

**Background:**

Increased scrotal temperature can disrupt spermatogenesis leading to male infertility. Germ cells being heat sensitive maintain their genetic integrity via protective mechanism originated from the cell itself or by means of cell death. However, qualitative differentiation of how reactive oxygen species and antioxidant enzymes regulate signaling pathways of cellular damage including DNA fragmentation at varied temperatures remains unexplored.

**Objective:**

The study was designed to evaluate the effects of heat mediated oxidative stress on male germ cells. Also, the time-dependent qualitative variation in the germ cell death was studied.

**Materials and Methods:**

Thirty male Wistar rats were randomly segregated into five major groups (n=6/each) i.e., Control, 30, 45, 60, and 90-min counterparts according to heat treatment protocol. Quantitation of DNA and DNA ladder studies was performed along with various biochemical parameter like lipid peroxidation (LPO), glutathione, catalase, superoxide dismutase (SOD), glutathione peroxidase (GSH-Px), glutatione reductase (GR), glutathione-s-transferase (GST).

**Results:**

Animals receiving heat treatment for 30-min and 45-min revealed systematic and gradual response to heat stress; whereas, 60-min and 90-min treated animals showed a typical and abrupt change of the internal milieu of germ cells. Laddering and smearing effect of damaged DNA in 30 and 45 min and 60 and 90 min heat treated animals was seen respectively.

**Conclusion:**

As the duration of heat treatment increases, the rate of apoptosis reaches an optimum level, and a further increase in the duration of heat treatment converted the mode of cell death from apoptosis to necrosis, implicitly due to severe oxidative attack.

## Introduction

Spermatogenesis is a complex, multi-step, temperature-dependent process that involves the proliferation and differentiation of spermatogonia into mature sperm (1). The male gonads require specialized temperature conditions in order to produce spermatozoa. The importance of thermoregulation in the testis is illustrated by the fact that the slight increase in scrotal temperature can disrupt spermatogenesis that ultimately can cause male infertility (2). Hence, it is sufficiently reasonable to state that there is an immense relationship between normal growth temperature and the body temperature in male germ cells. Numerous factors can elevate scrotal temperature, either by whole body or local scrotal heating. Elevated scrotal temperature has been found in men with febrile illness, retractile testes, occupations associated with high-temperature exposure, hot bath and sauna users and car drivers (3). 

Multiple human studies have also confirmed the deleterious effects of scrotal hyperthermia on spermatogenesis (4). Hyperthermia resulted in several detrimental effects on the testis, including DNA damage in germ cells as well as mature sperm along with a lengthy recovery period of the testis from mild transient heat shock (5). Apoptosis is a common process in spermatogenesis to sustain the genetic integrity and prevent abnormal spermatogenesis. The fact that scrotal hyperthermia increased germ cell apoptosis its contribution in the suppression of spermatogenesis is well marked (6). Reports suggest that apoptotic germ cells are first observed 8 hr after exposure at 43^o^C for 20 min. In contrast, a short exposure of the testes to lower temperatures of 39-40^o^C have no obvious effect on germ cell death (7). Also, it has been reported that male germ cells seem to have a threshold of time-temperature dosage for apoptosis (8, 9). 

Further, Kim and co-workers suggested the requirement of a time-temperature dosage for germ cell apoptosis (10). Various stresses and extracellular ligands generate and/or require reactive free radicals or derived species to successfully transmit their signal to the nucleus (11). In fact, many intracellular signalling pathways including those involving cell proliferation and death are known to be redox-sensitive. Low level of reactive oxygen species (ROS) is necessary for proper functioning of the cellular process including proliferation (12). At lower level oxidative attack induces apoptosis but at a higher level can cause necrosis (13, 14). 

In this direction, heat-induced ROS has been implicated as a mediator of germ cell death. However, it is not much clear whether ROS are a part of signal transduction cascade triggered by various inducers for apoptosis or if they are generated in parallel pathways that can independently trigger necrosis/apoptosis. Further, since the severity of damage to sperm cells subjected to heat stress varies with the intensity, frequency and duration of heat exposure indicating the possibility of conversion of apoptosis to necrotic germ cell death. It is important therefore to distinguish between ROS and antioxidant enzymes involved in such signalling pathways, and those that mediate general cellular damage including DNA fragmentation. There appears to be an important link between the oxidative and heat stress on male germ cells undergoing apoptosis. 

Consequently, it becomes more important to investigate how the different protocols of heat stresses affect the apoptosis and oxidative-reduction system. Currently, using an instant in vivo experimental model of induction of apoptosis in male germ cells after a single heat exposure, an attempt has been made to differentiate the signaling pathways and mechanisms of programmed germ cell death via qualitative analyses of ROS (15). 

The aim of the study was to evaluate the effects of heat-mediated oxidative stress on male germ cells and the time-dependent qualitative variation in the germ cell death.

## Materials and methods

**Animals**

In the current study, thirty male Albino rats (Wistar strain), 260-290 gr, procured from the Central Animal House, Panjab University, Chandigarh, India. The animals were housed in polypropylene cages bedded with sterilized rice husk and provided ad-libitum access to clean drinking water and standard animal pellet diet. Animals were acclimatized to departmental animal rooms for a period of one wk prior to the start of treatment. 

**Experimental design**

For the present investigations animals were randomly segregated into five major groups (n=6/each) i.e., control, 30, 45, 60, and 90 min counterparts according to the time period of heat treatment protocol. For the heat treatments, rats were anesthetized with an intraperitoneal (IP) injection of sodium pentobarbital (40mg/Kg body weight) and shifted to hyperthermic treatment apparatus. The rats were placed in thermostatically controlled water-bath at 43ºC in such a way that their scrotum containing testes got immersed in water for homogeneous hyperthermic exposure. The hyperthermic exposure was employed over different groups of animals for different time periods mentioned above. After exposures to heat treatment, animals were sacrificed immediately. The testes were extracted out, weighed carefully, and were processed for biochemical analyses and assessment of apoptosis.

**Assessment of apoptosis**

**DNA fragmentation analysis** **(DNA ladder Study)**

The DNA was isolated from freshly isolated testes by the organic method proposed by Adeli and Ogboma (16). Electrophoresis of DNA was carried out on 2.5% agarose gel (w/v) prepared in Tris-Borate-EDTA (TBE) buffer at pH=8.0. 

For analysis of DNA fragmentation by agarose gel electrophoresis, the DNA concentration of the samples was adjusted to approximately equivalent amounts. About 8 µg of DNA sample was mixed with loading buffer (10% glycerol and 0.025% bromophenol blue in water) and placed in the wells of agarose gel (2.5% w/v). The agarose gel placed in electrophoresis buffer TBE containing ethidium bromide was run initially at 2 Vcm^-1^ for few minutes till the samples moved into the gel and then at 7 Vcm^-1^ until the sample migrated to approximately 3 cm before the end of the gel. The gels were photographed under UV light with the help of gel documentation system and photographs were clicked for further analysis of electrophoretic patterns obtained.

**Spectrophotometric estimation of low**

**molecular weight (LMW) DNA content**

For estimation of DNA in 10,000×g supernatants, aliquots of the latter were treated with 2.5% tricholoro acetic acid (TCA) for protein precipitation. The mixtures were then centrifuged at 3000×g for 10 min and aliquots of the supernatants thus obtained were added to the Tris-EDTA (TE) buffer (1×) for taking the absorbance at 260 nm. 

**Biochemical analysis**

For Biochemical estimations, the testes were homogenized with a Potter Elvenjhem homogenizer and diluted to a concentration of 1g wet weight /4mL cold 50 mM Tris-HCl buffer (pH=7.4) containing 150 mM KCl and 0.25 M sucrose. Tissue homogenates were centrifuged at 10,000×g for 30 min at 4ºC and the supernatant was used for the estimation of the extent of oxidative stress by mean of lipid peroxidation (LPO) and glutathione (GSH) and enzymatic antioxidant defense mechanism. 

**LPO assay**

The levels of LPO were assayed using the method of Trush and co-workers (17). This method is based on absorption characteristics (absorption maxima at 532 nm) of TBA-MDA chromophore taken as an index of LPO. MDA levels were expressed as nanomoles of MDA per milligram of protein.

**Estimations of reduced Glutathione**

Estimation of GSH was performed in the tissue homogenates of testes by the method of Moron and co-workers (18). Reduced GSH is a non-protein sulfhydryl compound, 5, 5'-dithiobis (2-nitrobenzoic acid) (DTNB) is a disulfide compound which is readily reduced by sulfhydryl compounds, forming a highly colored yellow anion. The optical density of this yellow substance is measured at 412 nm. The assay was performed within 1 hr of sacrificing the animal so as to avoid errors due to oxidation of GSH. 100 l of 25% TCA was added to 500 l (125 mg tissue equivalent) of the homogenate. After mixing the contents, the precipitated proteins were separated by centrifugation at 2,000 g for 15 min. 

The supernatant was diluted in a test tube to 1.0 ml with 0.2 M sodium phosphate buffer, pH 8.0. To this, 2 ml of freshly prepared 0.6 mM 5, 5'-dithiobis (2-nitrobenzoic acid) (DTNB) in 0.2 M phosphate buffer, pH 8.0 were added. The optical density of yellow colored complex formed by the reaction of GSH and DTNB (Ellman’s reaction) was measured in double beam spectrophotometer at 412 nm against a reference which contained 0.1 ml of 0.2 ml of 5% TCA instead of the sample. Assay of known amounts of GSH in the presence of 0.1-0.2 ml of 5% TCA demonstrated that at this concentration it did not interfere with the GSH-DTNB complex formation. For each set of assays, a standard curve for GSH was prepared. All samples were run in duplicates.

**Catalase**

Catalase activity assay was done by the method of Luck, 1963 (19). The enzyme catalyzed the decomposition of hydrogen peroxide which was measured by a decrease in the absorbance at 240 nm. The activity was calculated using an extinction coefficient of 0.0394 mM^−1^ cm^−1^ and expressed as mM mole of H_2_O_2_ decomposed/min/mg protein. 

**SOD activity**

The activity of SOD was estimated in testes homogenates with the help of method as suggested by Kono (20). This method is based on the principle of the inhibitory effect of SOD on the reduction of nitroblue tetrazolium dye by superoxide anions, which were generated by the photo-oxidation of hydroxylamine hydrochloride (NH_2_OH.HCl). The activity of SOD was expressed as IU/mg protein, where 1 IU was defined as the amount of enzyme inhibiting the increase in OD by 50%. 

**Glutathione Peroxidase (GSH-Px)**

GSH-Px was assayed by the method of Paglia and Valentine (21). Using H_2_O_2_ as substrate. The activity was expressed as µmoles of reduced nicotinamide adenine dinucleotide phosphat (NADPH) oxidized/min/ mg protein. 

**Activity of Glutathione Reductase (GR)**

The activity of GR was determined by the procedure described by Carlberg and Mannervik (22). GR catalyses the reduction of oxidized glutathione to reduced GSH with the help of NADPH. The consumption of NADPH is directly related to the activity of GR. Enzyme activity was expressed as μmoles of NADPH oxidized/min/mg protein. 

**Glutathione-S-Transferase activity**

Assay of GST in 10000×g supernatant was done by the method as described by Habig and co-workers using 1-chloro-2, 4-dinitrobenzene as a substrate (23). 

**Protein estimation**

The protein content was measured according to the method of Lowry and co-workers (24).

**Ethical consideration**

This work was approved by the Research Degree Committee of Panjab University, Chandigarh, Union Territory, India (case no. 14870/Ph.D). The experimental protocols were performed in the Dept. of Zoology, Panjab University, Chandigarh and the care and treatment of animals were also in accordance with ethical guidelines issued by Panjab University, Chandigarh.

**Statistical analysis**

Statistical analysis was carried out employing Statistical Package for the Social Sciences (SPSS, version 10.00, SPSS Inc, Chicago, Illinois, USA) software. Data were expressed as Mean±SD for observation in each group. The statistical significance of inter group difference of various parameters was determined by unpaired student’s *t*-test. The statistical significance was depicted employing symbol "*" representing p<0.05. 

## Results

**DNA fragmentation**

**Figure 1:**
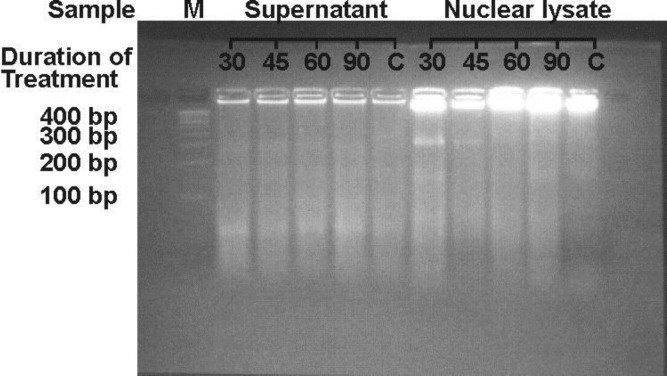
Electrophoresis of 10, 000g supernatant and nuclear lysate of germ cells in 2.5% agarose gel

**Figure 2:**
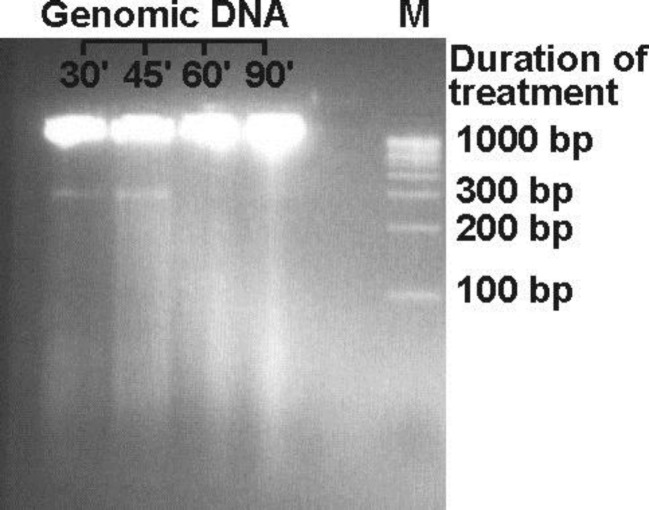
Electrophoresis of genomic DNA of male germ cells in 2.5% agarose gel. M-Marker, a hundred base pair ladder

Gel documents of agarose gel electrophoresis depicted the fragmentation pattern in the germ cells of all heat-treated animal groups. Electrophoretic pattern of 10,000×g supernatant from various continuous heat treatment experiments viz., 30, 45, 60, and 90 min, did not show any specific and clear fragmentation. Moreover, lysate of 30 and 45 min treated testes showed a highly intense 360 bp band and the intensity of 180 bp band was also found to be dominant. No clear bands were visible in 60 min heat treated animals. In lysate of 90 min as well as in control animals very faint bands of 180 and 360 bp were visible (Figure 1). Isolated DNA showed the characteristic band of apoptosis in 30 min as well as in 45 min continuous heat-treated animals but not visible in the other two groups. High molecular weight (HMW) DNA damage was the maximum in 60 min and 90 min continuous heat-treated animals when both compared to the previous group (Figure 2).

**LMW DNA content in 10,000×g supernatant**

**Figure 3:**
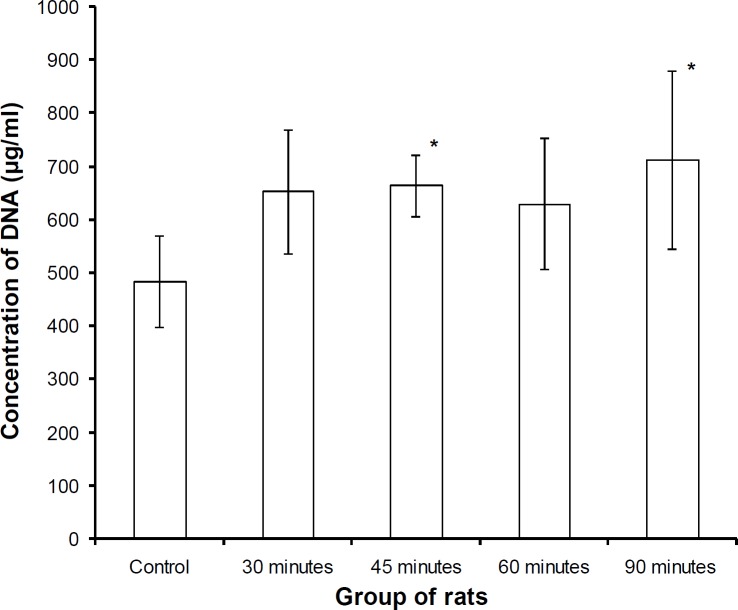
LMW DNA content in 10,000×g supernatant of testicular cells after various continuous heat treatment at 43˚ C in various animal groups

LMW DNA content in the supernatant of testicular homogenate was observed to increase gradually up to 45 min heat treatment, then sudden fall of LMW DNA content was observed in 60 min heat treated animals. Maximum LMW DNA content was observed in animals that received heat treatment for 90 min (Figure 3).

**Biochemical estimation**

A significant decrease in the extent of LPO was observed in the animals that received continuous heat treatment for 30 as well as 45 min as compared to the animals of the control group. However, in the animals that received continuous heat treatment for 60 and 90 min, the level of LPO was found to be increased. A non-significant alteration in the extent of GSH was observed in the animals that received continuous heat treatment when all compared to the animals of the control group. In the animals that received continuous heat treatment for 30 as well as 45 min, decreased levels of GSH were observed. However, in the animals that received continuous heat treatment for 60 and 90 min, the level of GSH was found to be increased (Table I). 

**Enzymatic antioxidant defense system**

Upon continuous heat treatment, alterations in the activity of catalase were observed when all heat-treated groups compared to control animals. In the animals that received the treatment for 30 as well as 90-min, the activity of catalase was observed increased significantly; however, in the animals that received heat treatment for 45 and 60 min decreased the activity of catalase in comparison to control animals was observed. The activities of SOD significantly higher in the animals that received heat treatment for 30, 45, and 60 min. The maximum increase in SOD was reported in the animals that received continuous heat treatment for 45 min. The values of SOD in the animals that received continuous heat treatment for 90 min were significantly lower than the control counterparts. The significant increase in the activity of GSH-Px was observed in 10,000×g supernatant of the testicular homogenate of animals that received heat treatment at 43^o^C for all the duration studied i.e., 30, 45, 60 and 90 min when all compared to control counterpart.

Moreover, the extent of change was found to be maximum in animals that received heat treatment for 30 min. Decreased activity of GR was observed in continuous heat-treated animal groups at 43^o^C for different duration studied when compared to control counterpart. The extent of change in the above said activities in the 10,000×g supernatant of testicular homogenate was minimum in the animals that received the treatment 60 min, whereas, the same was observed to be maximum in the animals that received treatment for 45 min, as compared to control ones.

Similar to GSH-Px, the GST activity was also observed to increase significantly in all treatment group studied viz., 30, 45, 60 and 90 min when all compared to control animals. Maximum activity of GST was found in animals that received continuous treatment for 60 min (Table II).

**Table I:** Lipid peroxidation and reduced glutathione content in control and continuous heat-treated testes after different time periods

**Estimation**	**Control**	**30 min**	**45 min**	**60 min**	**90 min**
LPO	1.87 ± 0.08	1.29 ± 0.15**	1.04 ± 0.14***	2.06 ± 0.25	1.90 ± 0.16
GSH	2.10 ± 0.23	1.83 ± 0.16	1.90 ± 0.48	2.14 ± 0.27	2.22 ± 0.10

Data presented as mean ± SD from 6 independent observations (n=6/each).

Symbols and statistical significance:

LPO: lipid peroxidation, n moles of MDA formed /minute /mg protein

GSH: glutathione, moles of GSH/mg protein

** and *** represent p ≤0.01 and 0.001, respectively when various heat treated animals compared to control counterparts

**Table II:** Specific activities of superoxide dismutase, catalase, glutathione peroxidase, glutathione reductase and glutathione-S-transferase in control and continuous heat-treatment experiment from 30 to 90 min at 43^o^C

**Specific Activity**	**Control**	**30 min**	**45 min**	**60 min**	**90 min**
SOD	31.84 ± 2.09	36.66 ± 4.25	50.41 ± 2.84***	37.77 ± 3.14*	25.63 ± 0.92**
CAT	11.22 ± 1.36	13.05 ± 0.70*	10.89 ± 0.75	8.67 ± 0.60*	14.48 ± 0.70**
GSH-Px	1.01 ± 0.27	2.88 ± 0.22***	1.65 ± 0.20**	1.77 ± 0.14*	2.30 ± 0.12***
GR	2.98 ± 0.36	1.73 ± 0.19***	1.41 ± 0.19***	2.85 ± 0.24	1.99 ± 0.08**
GST	2.26 ± 0.43	3.38 ± 0.41*	3.69 ± 0.27**	4.01 ± 0.26***	3.53 ± 0.65**

Data presented as mean ± SD from 6 independent observations (number of animal in each group=6). .

SOD: superoxide dismutase, International Unit

CAT: Catalase, moles H_2_O_2_ decomposed /minute /mg protein

GSH-Px: glutathione peroxidase, moles NADPH oxidized /minute/mg protein

GR: glutatione reductase, n moles NADPH oxidized/minute/mg protein

GST: glutathione-s-transferase, n moles GSH conjugates formed/minute/mg protein

*, ** and*** : p ≤0.05, p ≤0.01, and p ≤0.001 respectively when various heat treated animals compared to control counterparts

C- Control animals group, M- Marker, a hundred base pair ladder

Data presented as mean ± SD from 6 observations. *: p ≤0.05 when various heat -treated animals compared to control counterparts. Mean value is in g/ml of 10,000×g supernatants

## Discussion

In the current study, ROS mediated differential signalling pathways and mechanisms of germ cell death have been demonstrated after a single heat exposure. Exposure of mammalian cells to high temperatures, either in short-term (heat shock) or long-term hyperthermia, usually triggers several signalling pathways, some that facilitate cell survival and others that initiate cell death programs (25). It is proved that heating the scrotum has systemic effects as well as direct effects on the testes (26). In the mouse, direct effects of scrotal heating on the developing germ cells are reported to include altered synthesis of DNA, RNA and proteins, as well as protein denaturation and abnormal chromatin packaging (27). 

In the present study, the data of DNA fragmentation from agarose gel electrophoresis of 10,000×g supernatant of testes homogenate of rat testes that received treatment for 30, 45, 60 as well as 90 min indicate the absence of apoptotic cells. However, the nuclear lysate and isolated genomic DNA show the characteristic band of apoptosis in the animals that received heat treatment for 30 min and 45 min. This may be safely concluded that heat-induced/initiated the apoptosis in male germ cells after receiving treatment for up to 45 min. It may be due to initial stages of karyokinesis/karyolysis, LMW DNA was not released in the cytoplasm and also absent in the 10,000×g supernatant of the animals that received heat treatment for 30 and 45 min. Consistent with these findings, Kaur and coworkers recently reported induction of oxidative stress-mediated germ cell apoptosis during hyperthermic conditions with exposure duration as low as 30 min (28).

Agarose gel electrophoresis of 10,000×g supernatant, nuclear lysate and isolated genomic DNA of rat testes receiving heat treatment for 60 and 90 min did not show any specific bands of apoptosis; however, some smearing effect is visible. It may be due to that treatment of germ cell beyond the limit of apoptotic induction may convert apoptosis to necrosis or accidental cell death. Maximum damage in HMW DNA of 60 and 90-min heat treated animals, also supports this hypothesis. It may also be concluded that the formation of DNA ladder may appear at the time of apoptotic body formation and shifting the apoptotic cells into necrotic cells is the basis of failure in the ladder formation. Necrosis may be a substitute for failed apoptosis. Rapid apoptotic induction system shows early ladder with late smearing pattern of DNA in agarose gel (29). 

Several studies have shown that early morphological change of nuclear chromatin coincides with the appearance of HMW fragments, whereas the formation of the DNA ladder is a rather late event, occurring during or after apoptotic body formation has taken place (30-32). Heat stress induced by chronic exposure in rodents, a decrease in testicular weight and size of testes have been correlated with a decrease in diameter and necrosis of epithelial lining of seminiferous tubule (33). In the animals that received heat treatment for 60 min, the concentration of fragmented DNA in 10,000×g supernatant fell down to some extent, indicating a decline in the rate of apoptosis. Agarose gel electrophoresis also depicted the absence of characteristic bands of apoptosis. In the animals that received heat treatment for 90-min, the concentration of fragmented DNA slightly increased; however, the absence of characteristic bands of apoptosis may also support the hypothesis of conversation of the mechanism from apoptosis to the necrosis or accidental cell death. 

Takahashi and others also reported that heat-induced DNA fragmentation was detected with the comet assay (34). Moreover, Kampinga detected a slightly increased level of DNA fragmentation in heat-treated cells when compared with untreated cells using pulsed-field gel electrophoresis (35). These findings provide strong support for the idea that heat not only induced cell death but also induces DNA fragmentation. Lipid peroxidation is a very common phenomenon in stress and various pathological processes and also plays a significant role in the mechanism of cell death. The levels of LPO not corresponding with the increase in the rate of apoptosis in heat-induced oxidative stress might be due to the direct effect of heat on the induction of germ cells death. Increase in the duration of heat treatment up to 60 min and 90 min, slightly increased in the value in thiobarbituric acid reactive substances. Further, studies have shown that assays of peroxidation products do not always indicate oxidative damage (36, 37).

Free radicals may in part exert their effects without increasing peroxidation. Actually, metabolites of certain free radical-producing xenobiotics have been observed to inhibit the process of LPO (37, 38). Redox status inside a cell is crucial to the correct functioning of many enzymes and could act as a signalling mechanism (39). GSH is one of the most important redox-sensitive molecules and GSH depletion is associated with an increase in the rate of apoptosis and oxidative stress in various cell types. Currently, heat treatment slight decreases the GSH content in 10,000×g supernatant of testicular homogenate of 30 and 45 min heat treated animals which, might be due to the active involvement of GSH protection. Changes in GSH appear to affect apoptosis by regulating protein expression of members of the Bcl-2 family of protein (40-42). 

GSH depletion is associated with decreased Bcl-2 expression and increased apoptosis in cholangiocytes (43). The spermatogenic cells contain quite high levels of GSH. If the reducing power is too low, this would render spermatogenic cells prone to apoptosis by ROS and other stimuli (44). Dismutation of O2•ˉto H_2_O_2_ by SOD is often called the primary defense because this enzyme prevents the further generation of free radicals. The specific activity of SOD is directly correlated with O2•ˉ produced under the physiological condition in the cells. In the present study, during the continuous heat treatment for 30 and 45 min the value of SOD increases as the production of O2•ˉ increases. Loven and coworkers, also observed that SOD activity was increased at 1 and 8 hr after heat exposure, presumably coincidental with heat shock protein synthesis and suggest that SOD activity may be important in protecting cells exposed to heat and that it may play a role in the development of thermo-tolerance (45).

Further, an increase in the duration of heat treatment for 60 as well as 90 min, a decrease in the value of SOD was observed. This may be due to heat induce inactivation of this enzyme after continuous heat treatment. The activity of testicular SOD is also decreased in acute pro-oxidative conditions such as x-irradiation (46). Ahotupa and Huhxtaniemi also observed that the SOD activity in testicular cells and liver slices were inactivated by heat, and the testicular SOD was more sensitive. The initial increase in the activity of catalase in germ cell at 43^o^C for 30 min indicates the protective mechanism of the cells against heat stress (47). SOD/ Catalase ratio 60 as well as 90 min heat-treated group is drastically higher than the various other heat-treated animals which, might have led to an increase peroxidative attack. In the animals that received heat treatment for 90 min, the catalytic activity was observed maximum. Azevedo and coworkers, also suggested that the increase in the level of estrogen leads to an increase in the activity of catalase (48). 

GSH-Px is the general name of an enzyme family with peroxidase activity. It is very effective in converting H_2_O_2_ to water because the km of this enzyme for H_2_O_2_ is extremely low. With the increase in the temperature of male germ cell from the normal growth temperature to 43^o^C up to 30 min, increase in the peroxidase activities were observed which may be due to demand of cells to protect themselves from the time-dependent increase of the peroxide level. In the group of rats that received heat treatment continuously for 45 min, due to loss of huge population of cells the value of GSH-Px goes down. The activity of GSH-Px increase gradually from 45 min to 90 min heat treated testicular cells. The enhancement in the enzyme activity in heat treatment confirms the fact that GSH-Px is actively participating in protecting the cell from the toxicity of H_2_O_2_. GSH-Px in sperm is the major enzyme that removes H_2_O_2_ and protects cells against damage caused by free radicals and products of LPO in vivo (49). 

It is considered that GSH-Px to be a major protective system against the oxidative damage (50). Germ cells started receiving the heat treatment, the decline in the specific activity of GR has been observed. In necrotic cells, the GR activity is maximum as in the groups of 60 as well as in 90 min heat-treated animals this was due to increase in the level of oxidative stress. GSTs are basically phase-II enzymes of defense that and more potent in the genotoxic or cytotoxic condition of oxidative stress modulated by various electrophiles, carcinogen and genobiotics agents (51). These proteins serve in detoxification capacity to protect cells from various kinds of reactive substances (52) and the protective functions of GST are especially important for germ cells, in which electrophilic compounds would have a profound effect on sperm motility and are potentially hazardous to the integrity of DNA (53). 

Independent of various antioxidant studies the, level of GST was found elevated in the maximum heat-treated animals’ groups, which may be due to an increase in the genotoxic /cytotoxic condition of cells. Increase in GST activity was observed with increase in the duration of heat treatment. It may be due to heat dependent increase in the level of electrophilic or cytotoxic compound in the male germ cells. Hayes and Pulford (54) have suggested that the increase in oxidative stress due to ROS induce GST activity. Cells apoptosis induced by intense heat stress is the prominent feature of heat-related illness and antioxidant significantly decreased the heat stress (55). They also found that generation of reactive oxygen species (ROS) was a critical mediator in heat stress-induced apoptosis. 

Present observation directly indicates that there is a different type of stress in animals group that received heat treatment for 30 min and 45 min and other two animals group that received heat treatment for 60 min and 90 min. Animals that receive heat treatment for 30 min and 45 min reveal a gradual response of heat stress. On the other hands, 60 min, as well as 90 min, treated animals reveal the typical and abrupt changes in the internal milieu of male germ cells due to oxidative attack, which is also a potent reason of conversion of apoptosis into necrosis in male germ cells.

## Conclusion

As the duration of heat treatment increases, the rate of apoptosis enhances up to an optimum, however, with further increase in the duration of heat treatment mode of cell death is converted from apoptosis to necrosis (sudden death). It is also suggested that heat has a direct effect on male germ cell death. It is clear from the estimation of LPO, GSH and various antioxidant enzymes that differential qualitative stress exists between 30 and 45 min and 60 and 90 min treatment.
